# Multiple huge epiphrenic esophageal diverticula with motility disease treated with video-assisted thoracoscopic and hand-assisted laparoscopic esophagectomy: a case report

**DOI:** 10.1186/s40792-017-0339-6

**Published:** 2017-05-08

**Authors:** Yoshiki Taniguchi, Tsuyoshi Takahashi, Kiyokazu Nakajima, Shigeyoshi Higashi, Koji Tanaka, Yasuhiro Miyazaki, Tomoki Makino, Yukinori Kurokawa, Makoto Yamasaki, Shuji Takiguchi, Masaki Mori, Yuichiro Doki

**Affiliations:** Department of Gastroenterological Surgery, Osaka University Graduate School of Medicine, 2-2 Yamadaoka, Suita, Osaka 565-0871 Japan

**Keywords:** Epiphrenic esophageal diverticulum, Esophageal motility, Hypertensive lower esophageal sphincter, Video-assisted thoracic surgery

## Abstract

**Background:**

Epiphrenic esophageal diverticulum is a rare condition that is often associated with a concomitant esophageal motor disorder. Some patients have the chief complaints of swallowing difficulty and gastroesophageal reflux; traditionally, such diverticula have been resected via right thoracotomy. Here, we describe a case with huge multiple epiphrenic diverticula with motility disorder, which were successfully resected using a video-assisted thoracic and laparoscopic procedure.

**Case presentation:**

A 63-year-old man was admitted due to dysphagia, heartburn, and vomiting. An esophagogram demonstrated an S-shaped lower esophagus with multiple epiphrenic diverticula (75 × 55 mm and 30 × 30 mm) and obstruction by the lower esophageal sphincter (LES). Esophageal manometry showed normal peristaltic contractions in the esophageal body, whereas the LES pressure was high (98.6 mmHg). The pressure vector volume of LES was 23,972 mmHg^2^ cm. Based on these findings, we diagnosed huge multiple epiphrenic diverticula with a hypertensive lower esophageal sphincter and judged that resection might be required. We performed lower esophagectomy with gastric conduit reconstruction using a video-assisted thoracic and hand-assisted laparoscopic procedure. The postoperative course was uneventful, and the esophagogram demonstrated good passage, with no leakage, stenosis, or diverticula.

**Conclusions:**

The most common causes of mid-esophageal and epiphrenic diverticula are motility disorders of the esophageal body; appropriate treatment should be considered based on the morphological and motility findings.

## Background

Esophageal diverticula are rare structural abnormalities of the esophagus and are categorized by their location and etiology. An epiphrenic diverticulum is a pulsion diverticulum in which the mucosa and submucosa herniate through the muscular layers in the distal 10 cm of the esophagus [1]. Previous studies have shown that in the majority of patients (75–100%), epiphrenic diverticula were associated with esophageal achalasia or other esophageal motility disorders, such as diffuse esophageal spasm or a nutcracker esophagus [2–5]. Here, we report a case with huge multiple epiphrenic diverticula with a hypertensive lower esophageal sphincter (HLES), who was successfully treated with video-assisted thoracic surgery-esophagectomy (VATS-E) with gastric conduit reconstruction by hand-assisted laparoscopic surgery (HALS).

## Case presentation

The patient was a 63-year-old man who presented with the chief complaints of swallowing difficulty, heartburn, and vomiting. Although he had begun to notice those symptoms 10 years previously, the condition had been followed up conservatively. He had noticed worsening of the swallowing difficulty, had also developed vomiting, and therefore visited a hospital near his home. Upper gastrointestinal endoscopy revealed two lower esophageal diverticula on the right and left walls, and he was finally referred to our hospital in December 2014. His medical history included hypertension and bronchial asthma; there was no history of smoking or drinking.

**Fig. 1:**
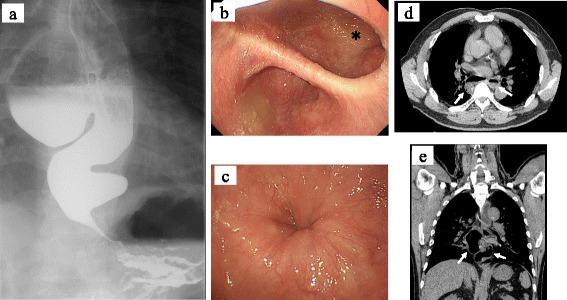
Preoperative imaging findings. **a** An upper gastrointestinal contrast study demonstrates dilatation of the esophagus and epiphrenic diverticula. **b** Upper gastrointestinal endoscopy shows a huge diverticulum in the lower esophagus (*asterisk*). **c** Endoscopic examination showed that the rosette-like esophageal folds appeared in the lower esophagus. **d**, **e** Chest computed tomography demonstrates dilatation of the esophagus and the epiphrenic diverticula of 50 × 40 mm and 25 × 25 mm in size. *Arrows* indicate the diverticula

**Fig. 2:**
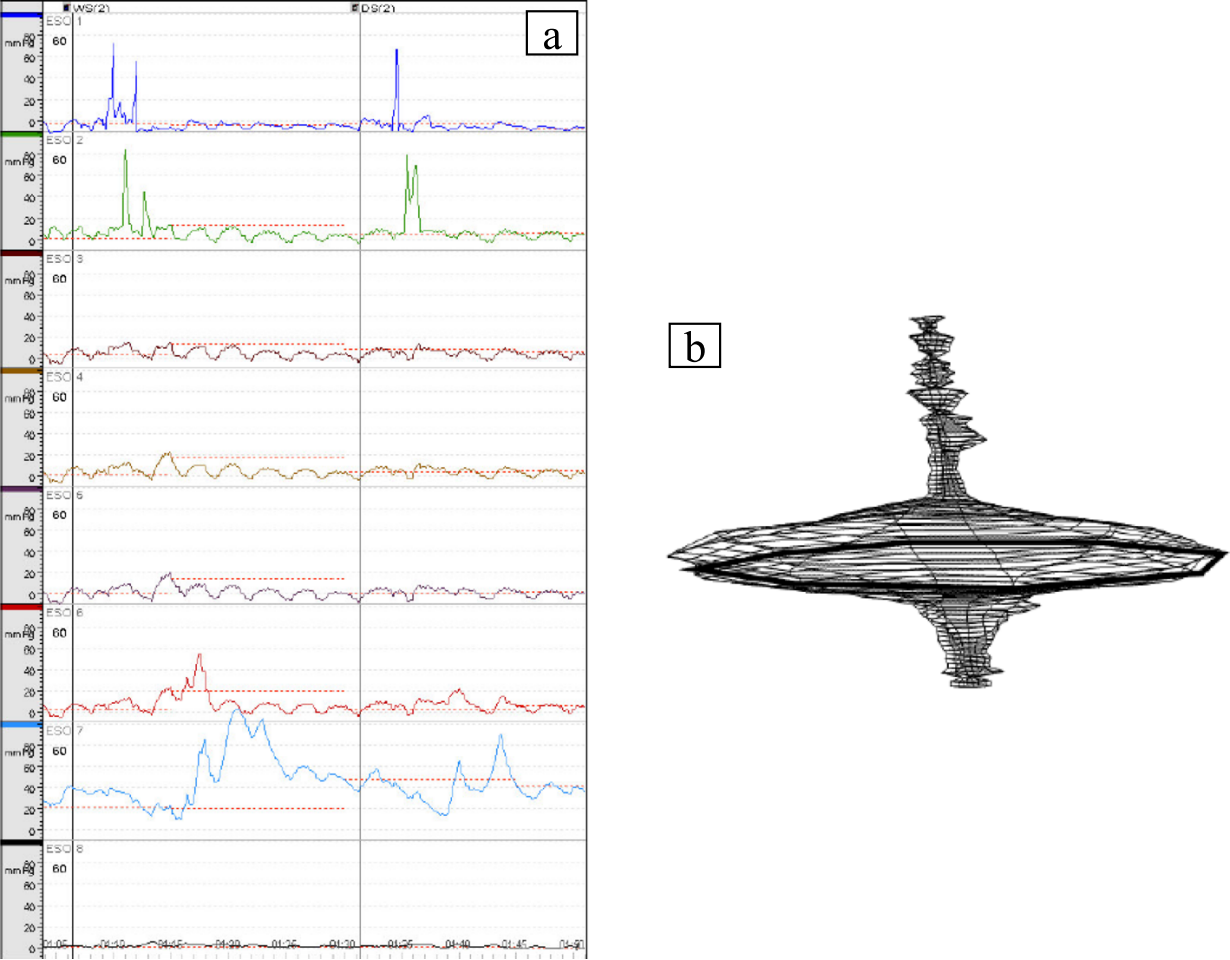
Preoperative esophageal manometry findings. **a** Intra-esophageal pressure measurement showing a high lower esophageal sphincter pressure and normal peristaltic waves. **b** The vector volume is very high (23,972 mmHg^2^ cm)

Upon admission, physical examination of the patient was completely normal. Laboratory findings, including complete blood count, erythrocyte sedimentation rate, and biochemical tests, were all within normal limits. An upper gastrointestinal contrast study revealed two diverticula with diameters of 7.5 × 5.5 cm and 3 × 3 cm on the right and left walls, respectively. The entrances of the diverticula were located in the lower esophagus, and the lower esophagus was sigmoid-shaped, and pooling of barium in the diverticulum, delayed passage into the stomach, and dilation of the esophagus were seen (Fig. 1a). Upper gastrointestinal endoscopy revealed the inlet of the diverticulum on the right wall of the lower esophagus at a distance of 34 cm from the incisors and another diverticulum immediately above the esophagogastric junction at a distance of 40 cm from the incisors (Fig. 1b). The esophagogastric junction was not well opened by air supply (Fig. 1c). A chest computed tomography (CT) scan showed food debris and fluid collection within the diverticula (Fig. 1d, e). Intra-esophageal pressure measurement showed a high lower esophageal sphincter (LES) pressure of 98.6 mmHg, with a length of 29 mm, a vector volume of 23,972 mmHg^2^ cm, and normal peristaltic waves (Fig. 2a, b). In addition, LES relaxation was not noted during the manometry test, but it was observed during swallowing.

**Fig. 3:**
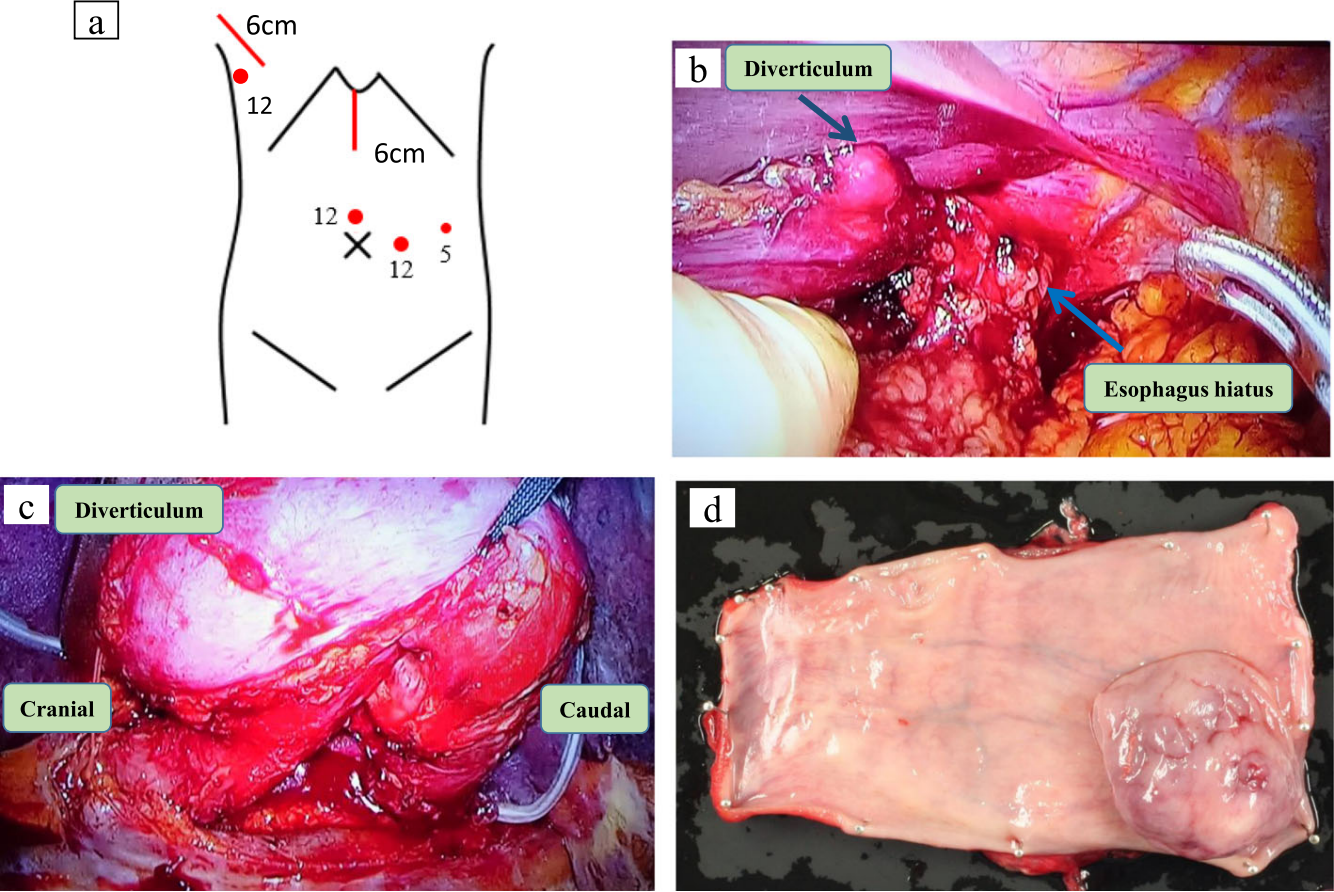
Operative findings and resected specimen. **a** The skin incision and the trocar placement (two 12-mm trocars and one 5-mm trocar in the abdomen and one 12-mm trocar in the chest). **b** Peeling of the hiatus. **c** Taping the huge right-sided diverticulum. **d** Resected specimen showing no abnormalities on its mucosal surface

Based on these findings, the patient was diagnosed with huge diverticula with HLES. Surgery was performed because of the chronic symptoms. First, the abdominal procedure was performed in a supine position. A 6-cm upper-middle abdominal incision was made, and two 12-mm trocars were inserted above and to the left side of the umbilicus, and a 5-mm trocar was inserted on the left side of the body (Fig. 3a). While performing the HALS technique, after peeling off the esophageal hiatus, the left-sided diverticulum was identified in the lower part of the esophagus (Fig. 3b). The stomach was transected under the esophagocardial junction using a linear stapler. A sub-total gastric conduit was made using a linear stapler outside the abdominal wall. The abdominal esophagus and the sub-total gastric conduit were joined together by using sutures. Next, the patient was positioned in a left lateral position, one 12-mm trocar was inserted into the sixth intercostal space, and thoracotomy was performed at the level of the fourth intercostal space. After identifying and taping the huge right-sided diverticulum (Fig. 3c), the esophagus was resected immediately above the upper diverticulum, and the specimen was removed. The gastric conduit was lifted up via the posterior mediastinal route under the surgeon’s manual guidance, and the esophagogastrostomy was completed using a circular stapler. The surgical time was 295 min, and the intraoperative blood loss was 260 ml. Macroscopically, there were no abnormalities observed on the mucosal surface of the resected tissue (Fig. 3d).

The postoperative course was uneventful, and an upper gastrointestinal contrast study demonstrated good passage, with no leakage or stenosis. At 8 months after the surgery, the symptoms had disappeared, and a recent upper gastrointestinal contrast study also demonstrated no recurrence of the diverticula and good passage.

### Discussion

Epiphrenic esophageal diverticula are often associated with an underlying motility disorder of the esophagus, such as achalasia and achalasia-related diseases [6–10]. These diseases indicate a functional disorder with an unknown cause. Consequently, the esophageal passage is often obstructed, and esophageal dilation is commonly observed [7]. Therefore, evaluation of esophageal motility is a very important factor in assessment of this condition; however, esophageal manometry has rarely been reported in this context [8, 9]. In the present case, we evaluated not only morphological findings from an upper gastrointestinal contrast study and endoscopic study but also esophageal functional evaluation by esophageal manometry. The latter revealed high levels of pressure on the LES, normal peristaltic contractions, and LES relaxation. Based on these findings, we diagnosed HLES, which is one of the esophageal achalasia-related diseases; and HLES might induce the epiphrenic esophageal diverticula. In addition, although most patients with esophageal diverticula are asymptomatic [10], patients with motility disorders often complain of swallowing difficulty and the symptoms of gastroesophageal reflux [11, 12].

Since medical and endoscopic therapies play limited roles in treatment, surgery is the standard treatment for patients with incapacitating symptoms related to an epiphrenic esophageal diverticulum, such as dysphagia, regurgitation, aspiration, pneumonia, heartburn, and chronic coughing [13, 14]. Although the size of the diverticulum is not an indication for surgery, spontaneous rupture has been documented in a few patients with large diverticula [15]. Traditionally, diverticulectomy, myotomy, and fundoplication have been reported as standard treatment methods [16]. However, postoperative staple line leakage, caused by the increase of the intra-esophageal pressure, has been reported to occur in 7.7–27.2% after diverticulectomy and myotomy for esophageal diverticula [17–19]. In the present case, the diverticula were huge and multiple; moreover, the esophagus had an S-shaped dilatation due to the chronic high intra-esophageal pressure. Therefore, we considered that diverticulectomy might cause staple line leakage and that myotomy would not improve the food stagnation. A lower esophagectomy was thought to be the better surgical method for achieving cure.

Up to the 1990s, surgery was typically performed using the transthoracic approach through a right thoracotomy (most diverticula arise from the right side of the esophagus) [20–22]. With advances in minimally invasive operative techniques [23–25], laparoscopy has also become a reasonable alternative to open surgery, and laparoscopic diverticulectomy with myotomy and fundoplication are now considered to be the approach of choice in most cases [5, 9, 26]. Although there have been some case reports of epiphrenic esophageal diverticula that were resected laparoscopically [27, 28], almost the entire diverticulum could be visualized laparoscopically from the esophageal hiatus by pulling the esophagus caudally. In the present case, although the left-sided diverticulum could be identified by a transhiatal approach, the huge diverticulum present in the upper part of the esophagus could not be observed. To resect both diverticula, both a thoracic and an abdominal approach were necessary. On the other hand, HALS has the advantage of allowing better visualization and is useful for the transhiatal procedure by avoiding the liver, pulling the esophagus, as well as for protecting the stomach. We have employed the HALS technique for gastric conduit reconstruction in esophageal surgery [29]. In our experience, postoperative complications were significantly less in HALS than in open surgery when performing esophageal cancer surgery with gastric conduit reconstruction.

## Conclusions

In conclusion, we here reported a case with multiple epiphrenic esophageal diverticula with HLES, who was successfully treated by VATS-E using gastric conduit reconstruction by the HALS technique. It is important to consider the appropriate treatment of complicated epiphrenic esophageal diverticula when deciding on surgery to cure this condition.

## Abbreviations

HALS: Hand-assisted laparoscopic surgery
HLES: Hypertensive lower esophageal sphincter
LES: Lower esophageal sphincter
VATS-E: Video-assisted thoracic surgery-esophagectomy
